# Impact of an alternating first-line antibiotics strategy in febrile neutropenia

**DOI:** 10.1371/journal.pone.0208039

**Published:** 2018-11-28

**Authors:** Ban Hock Tan, Marvin Raden Torres De Guzman, Lara Kristina Sioco Donato, Shirin Kalimuddin, Winnie Hui Ling Lee, Ai Ling Tan, Gee Chuan Wong

**Affiliations:** 1 Department of Infectious Diseases, Singapore General Hospital, Singapore, Singapore; 2 Department of Haematology, Singapore General Hospital, Singapore, Singapore; 3 Department of Pharmacy, Singapore General Hospital, Singapore, Singapore; 4 Department of Microbiology, Singapore General Hospital, Singapore, Singapore; Rabin Medical Center, Beilinson Hospital, ISRAEL

## Abstract

**Background:**

Rising antibiotic resistance poses a challenge to the management of febrile neutropenia in patients with haematological malignancies receiving chemotherapy.

**Aim:**

We studied an alternating first-line antibiotic strategy to determine its impact on all-cause mortality and bacteremia rates in patients with febrile neutropenia.

**Methods:**

An alternating first-line antibiotic strategy was established in mid-2013. Data for 2012 (before strategy implementation) and 2014 (post-strategy implementation) were compared. Antibiotic Heterogeneity Index (AHI) for each of the two time-periods was also calculated.

**Findings:**

There were 2012 admissions (26082 patient-days) in 2012 and 1843 admissions (24331 patient-days) in 2014. There was no significant difference in the baseline characteristics of patients in the two groups. The defined daily doses (DDD) of cefepime (CEF) fell while the DDD of piperacillin-tazobactam (PTZ) rose in 2014 compared with 2012. Vancomycin DDD fell in 2014. The AHI was 0.466 in 2012 and 0.582 in 2014. The difference in all-cause mortality was not statistically significant. There was no difference in rates of bacteremia with CEF-resistant, PTZ-resistant and carbapenem-resistant gram-negative organisms in the two groups. Rates of new cases of Methicillin-resistant *Staphylococcus aureus* (MRSA) were 2.38/1000 and 2.59/1000 patient-days in 2012 and 2014 respectively. Rates of new cases of Vancomycin-resistant *Enterococcus* (VRE) were 1.84/1000 and 1.81/1000 patient-days in 2012 and 2014 respectively. There was no Carbapenem-resistant Enterobacteriaceae (CRE) bacteremia in 2012 and 1 in 2014.

**Conclusion:**

An alternating first-line antibiotic strategy resulted in an increase in antibiotic heterogeneity, without increasing mortality. There was also no significant increase in bacteremia rates.

## Introduction

Empirical antibiotics are crucial in the management of fever in patients who are neutropenic after chemotherapy for haematological malignancies. [1]

Rising antibiotic resistance, however, threatens to limit the options for empirical and definitive treatment. Infections with multi-drug resistant organisms (MDROs) carry a high mortality, especially in the haematology-oncology setting. [2–4] Of particular concern are the ESKAPE organisms (*Enterococcus faecium*, *Staphylococcus aureus*, *Klebsiella pneumoniae*, *Acinetobacter baumannii*, *Pseudomonas aeruginosa and Enterobacter* species) which are the leading cause of nosocomial infections among immunocompromised individuals and are often characterized by antibiotic resistance. [5]

Judicious antibiotic use, generally through an antibiotic stewardship program, is recommended as part of a multipronged approach against MDROs. [6] Like other haematology units, we were concerned about the increase in multi-, extremely- and pan-drug resistant (M-, X-, P-DR) gram-negatives, and their adverse impact on patient survival.[2, 3, 7–9]

Antibiotic cycling has been mooted as one way in which the development of resistance may be prevented. [10] The principle behind cycling is straightforward. Heavy use of an antibiotic over a prolonged period will likely engender resistance to that antibiotic. It is posited that a scheduled withdrawal of that antibiotic (or all in its class) for a set period should re-establish susceptibility to that antibiotic. During the period when the original antibiotic is restricted, a suitable alternative is used. [10, 11] As this alternative has not been used in the previous period, most of the organisms in the unit should be susceptible to it. The process can be repeated–hence the term cycling.

Unfortunately, the results of cycling studies have been mixed. In a bone marrow transplant unit, cycling was associated with stable rates in gram-negative resistance but a rise in ampicillin and vancomycin resistance among Enterococci. [12] In another study, cycling did not increase gram-negative resistance, but there was an increase in VRE. [13] Outside of the Haematology-Oncology setting, attempts at cycling have also yielded modest results at best. [10] Methodological flaws, the use of historical controls and concomitant interventions all make conclusions difficult. [11]

In addition to antibiotic cycling, mathematical models have shown that heterogeneous antibiotic use (i.e. a balanced use of antibiotics) could reduce the emergence of resistance.[11] A strategy of antibiotic “mixing” was attempted by Sandiumenge *et al* in an ICU. [14] During periods of high antibiotic homogeneity, the rates of carbapenem-resistant *Acinetobacter baumannii* and extended spectrum beta-lactamase (ESBL)-producing Enterobacteriaceae were high, but fell during the more heterogeneous period. [14] In a repeat study that focused only on the ESKAPE organisms, Sandiumenge *et al* again showed that during the “mixing” period, there was a lower rate of resistant ESKAPE organisms. [15] A Japanese study also showed that increasing antibiotic heterogeneity reduced the incidence of gram-negative infections. [16]

Our hospital is a 1700 bed tertiary hospital. In the Department of Haematology (DoH), cefepime (CEF) had been the first-line empirical antibiotic in febrile neutropenia for several years. [17]

We hypothesized that antibiotic heterogeneity might retard the inexorable march towards pan-drug resistance, without significantly affecting mortality.

## Methods

### Strategy

**Fig 1:**
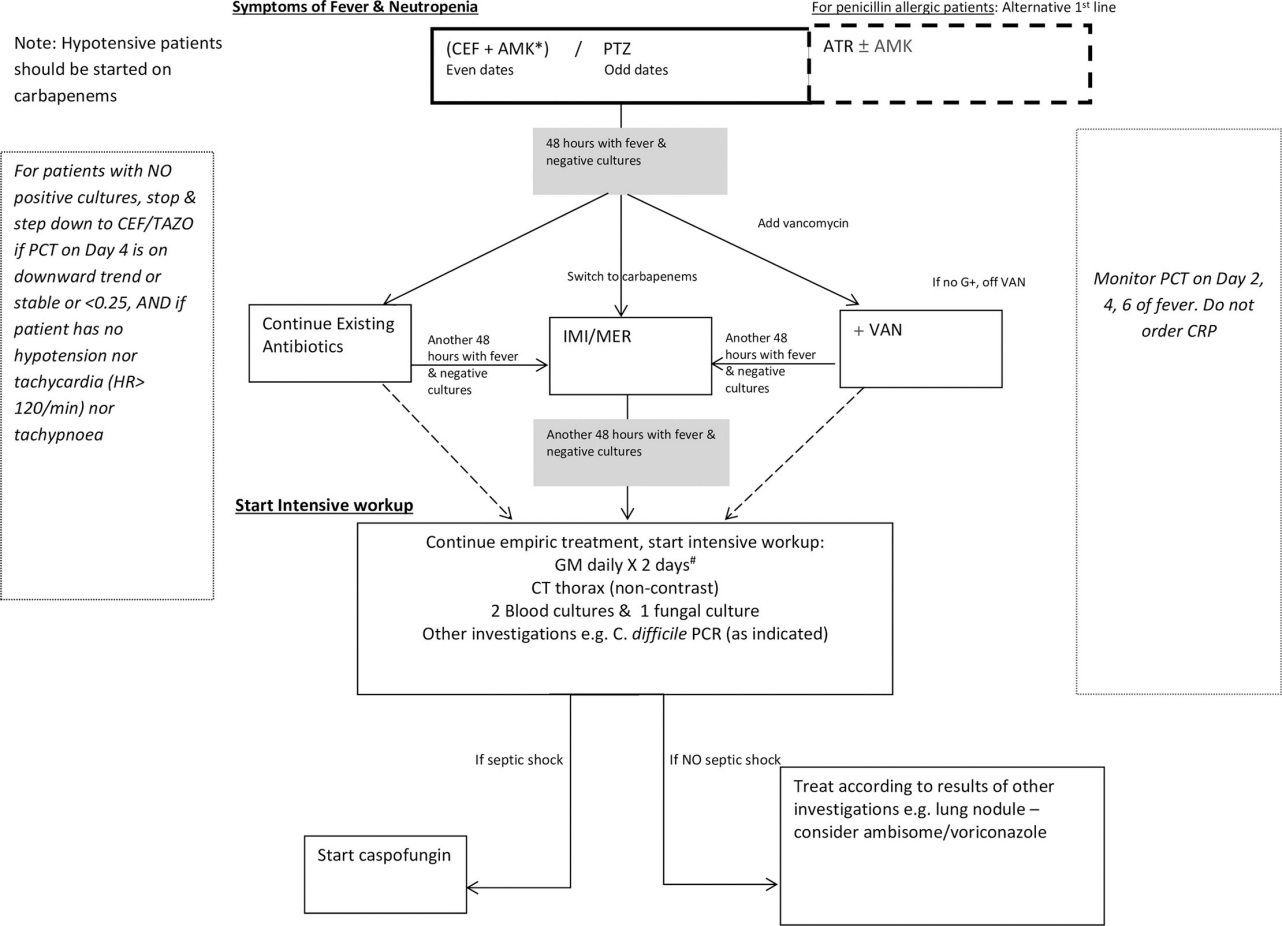
Workflow for empiric treatment of febrile neutropenia in haematology unit. **Abbreviations:** ATR-Aztreonam, AMK-Amikacin, CEF-Cefepime, GM-Galactomannan, CRP- C-reactive protein, G+-Gram positive bacteria cultured, HR–Heart rate, IMI-Imipenem, MER-Meropenem, PCT-Procalcitonin, VAN-Vancomycin, PTZ- Piperacillin-tazobactam, PCR- Polymerase Chain Reaction. *Check amikacin levels, consider stopping after 72 hours if cultures negative & patient is stable # then test GM levels weekly till Absolute Neutrophil Count >1000. ————Alternative strategies in workflow.

In view of data negative for cycling and promising for antibiotic heterogeneity, increasing antibiotic heterogeneity was considered a viable approach to the rising problem of MDROs. After a consensus meeting comprising haematologists and infectious diseases physicians, it was decided that the choice of empiric (i.e. first-line) antibiotic for febrile neutropenia would be piperacillin-tazobactam (PTZ) on odd dates, and a cefepime-amikacin (CEF-AMK) combination on even dates. (See Fig 1) Patients with a beta-lactam allergy would be started on aztreonam. If the patient was still febrile at 48 hours, and cultures remained negative, physicians had several options, including continuing the originally prescribed antibiotics, adding vancomycin, or switching to a broad-spectrum carbapenem [either imipenem (IMI) or meropenem (MER)]. This strategy of alternating first-line antibiotics was implemented in mid-2013.

We embarked on this before-and-after study to assess the utility and consequences of this use of alternating first-line antibiotics in febrile neutropenia. The primary objective was to determine if there was any impact on all-cause mortality in 2014 (after strategy implementation) compared to 2012 (before strategy implementation). The secondary objectives were to determine differences in rates of the following between the two study periods: bacteraemia-associated deaths, new cases of MRSA, new cases of VRE, CEF-resistant Gram-negative bacteraemia, PTZ-resistant Gram-negative bacteraemia and carbapenem-resistant Gram-negative bacteraemia. “New cases” of MRSA and VRE included those infected with these organisms, as well those who were colonized.

We collected data on baseline characteristics of patients, disease-type and clinical outcome for the years periods 1 January 2012–31 December 2012 and 1 January 2014–31 December 2014, for two Haematology wards (total 52 beds). In addition, we also collected the following data for these two periods: bacteraemia (including rates of bacteraemia caused by carbapenem-resistant organisms), new cases of MRSA, new cases of VRE and mortality. We calculated the incidence density of carbapenem-resistant Enterobacteriaceae (CRE) in patients admitted to the DoH and compared it with the rest of the hospital. All microbiology data were obtained from the hospital’s Laboratory Information System, and individually reviewed by the investigators. The defined daily doses (DDD) of CEF, PTZ, IMI and MER for 2012 and 2014 were calculated. All drug use data were obtained from the Pharmacy. The Antibiotic Heterogeneity Index (AHI) was calculated according to the formula used by Sandiumenge *et al*. [14] Data on admissions, discharge diagnoses and mortality were obtained from the hospital’s data warehouse, with the help of the Information Technology office. Every death occurring in a bacteraemia patient was reviewed by the two senior authors to establish if there was a link between bacteraemia and death, following the guidelines issued by the US Department of Health and Human Services. [18] This study was approved by the SingHealth Centralized Institutional Review Board (CIRB Ref 2013/824/F) with a waiver for informed consent.

### Clinical and infection control practices

In the DoH, ciprofloxacin prophylaxis is provided routinely for all patients expected to experience prolonged neutropenia. Blood cultures will be drawn, and antibiotics may be commenced or adjusted when a neutropenic patient spikes a fever. Blood cultures drawn from each patient consist of two aerobic and two anaerobic bottles (BACTEC Plus Aerobic/F and BACTEC Plus Anaerobic/F respectively), as well as one dedicated fungal bottle (BACTEC Myco/F lytic), from at least two different sites. These were not changed in the two periods of the study.

All new admissions are screened for MRSA (nose, axilla, and groin) and VRE (rectal). From August 2014, DoH admissions were also screened for CRE via rectal swabs.

Due to a limited number of single rooms, single rooms are only used to house the following categories of patients: MRSA respiratory dispersers, VRE-positive patients with diarrhoea and patients with *C*. *difficile* diarrhoea (for up to two days after diarrhoea stops). Patients who test positive for a respiratory virus are isolated in single rooms. These were not changed in the two periods of the study.

### Laboratory methods

Please refer to S1 Appendix for details.

### Definitions

Fever was defined as a single temperature measurement of >38.3°C (101°F) or a temperature of >38.0°C (100.4°F) sustained over a 1-hour period.[1] Neutropenia was defined as an absolute neutrophil count (ANC) of <500 cells/mm^3^ or an ANC that was expected to decrease to <500 cells/mm^3^ during the next 48 hours. [1] A single positive blood culture was adequate for the definition of bacteraemia, except for growth of coagulase-negative *Staphylococcus*, *Bacillus*, *Corynebacterium* or other skin contaminants, for which growth from at least two bottles, taken at different times, was needed. New cases of MRSA or VRE was defined as the isolation of that organism from either clinical or screening specimens in a patient who had never been positive previously, if the isolate grew from a sample submitted ≥48 hours after hospital admission. For multiply-admitted patients, a positive admission screen made the patient a new case, if previous admission screens were negative.

### Statistical methods

Baseline characteristics of the two groups were summarized using descriptive statistics and the chi-square test was used to compare non-continuous variables. Antibiotic usage was expressed as DDD (defined daily doses) per 1000 patient days. The Poisson distribution method was used to calculate the 95% confidence interval (CI) for the incidence rate ratio of bacteraemia, new cases of MRSA and VRE between the two groups. A p-value of <0.05 was considered statistically significant.

## Results

In 2012, there were 2012 admissions (26082 patient days) while in 2014 there were 1843 admissions (24331 patient days) to the two wards under study. The baseline characteristics of the patients admitted in these two different years are shown in Table 1.

**Table 1:** Baseline characteristics.

Baseline Characteristics	Year 2012 n	Year 2014 n	p-value^a^
Total admissions	2012	1843	-
Median Age (Range)	58 (12–97)	60 (14–95)	-
% of Males	54.5	55.7	0.58
Adm with New AML	93	55	**0.004**
Adm with New ALL	24	14	0.15
Adm with AML- on therapy^b^	60	43	0.19
Adm with ALL- on therapy^b^	15	9	0.31
Adm with lymphoma	341	296	0.46
Adm with myeloma	95	113	0.054
Adm with MDS	69	68	0.66
Mean LOS (days) Ward A Ward B	9.7 10.6	10.0 11.6	-

Abbreviations: Adm—admissions, AML—acute myeloid leukaemia, ALL—acute lymphocytic leukaemia, MDS—myelodysplastic syndrome, LOS-Length of Stay

^a^ Calculated by chi-square test

^b^ Refers to patients who had treatment for the disease in our unit, specifically induction, consolidation and salvage treatment only.

Although there were more new diagnoses of acute myeloid leukaemia (AML) in 2012, the number of patients on chemotherapy for AML in 2012 was not different statistically from 2014. There was no difference in the number of admissions for the other malignant conditions, e.g. myeloma. (Table 1) The spread of non-malignant diagnoses (e.g. thalassemia, autoimmune haemolytic anaemia) was also not different.

In 2014, the DDD of CEF fell (235.25/1000 patient days in 2012 vs. 108.82/1000 patient days in 2014, p<0.001), and the DDD of PTZ rose (20.73/1000 patient days in 2012 vs. 86.05/1000 patient days in 2014, p<0.001). (Table 2) The DDD of vancomycin fell in 2014 compared with 2012 (116.62/1000 patient days in 2012 vs. 70.82/1000 patient days in 2014, p<0.001).

**Table 2:** Antibiotic use in the 2 years under study.

Antibiotic used	Year 2012 DDD /1000 patient days	Year 2014 DDD /1000 patient days	p-value ^a^
Aminoglycosides^b^	43.46	37.73	0.58
Aztreonam	27.38	21.68	0.48
Carbapenems	236.78	211.35	0.22
Cefepime	235.25	108.82	**<0.001**
Ciprofloxacin	91.57	68.89	0.08
Meropenem	228.03	207.23	0.82
Polymyxin	21.10	25.18	0.56
Piperacillin-tazobactam	20.73	86.05	**<0.001**
Vancomycin	116.62	70.82	**<0.001**
Linezolid	20.44	6.80	**<0.001**
Daptomycin	5.50	14.92	**<0.001**
Gram-positive agents^c^	142.56	92.53	**<0.001**
All antibiotics	1616.86	1250.50	**0.029**

Abbreviations: DDD-defined daily dose

^a^ Calculated by chi-square test

^b^ Total of amikacin & gentamicin

^c^ Total of vancomycin, linezolid and daptomycin

The rate of use of other antibiotics was not different statistically. The AHI was 0.466 in 2012 and 0.582 in 2014. There was no change in the rates of new MRSA and new VRE cases in the years 2012 & 2014. (Table 3) The difference in the primary end-point (all-cause mortality) was not statistically significant: 11% in 2012 vs. 9% in 2014 (p = 0.12). (Table 4) Overall rates of bacteraemia were similar in both years, as were the rates of bacteraemia due to gram-negative organisms. (Table 5) There was no difference in the rate of isolation from blood cultures of CEF-resistant, PTZ-resistant and carbapenem-resistant gram-negatives in the 2 years under study. (Table 5) Since CRE screening for Haematology patients only started in mid-2014, there are no Haematology-specific rates for comparison. However, the hospital-wide rates were 0.05/1,000 patient days and 0.25/1,000 patient days in 2012 and 2014 respectively, for hospital-onset CRE. In terms of infection rates, there was no CRE bacteraemia in the two wards under study in 2012, and 1 in 2014 (p = 0.48, Table 5).

**Table 3:** New cases of MRSA & VRE in the 2 years under study.

New Cases of MRSA and VRE	Year 2012 N = 2012 n	Year 2014 N = 1843 n	Incidence Rate Ratio (95% CI) ^a^	p-value ^b^
MRSA new, n	62	63	**-**	**-**
MRSA new /1000 patient days	2.37	2.59	1.089 (0.77, 1.55)	0.697
VRE new, n	48	44	**-**	**-**
VRE new /1000 patient days	1.84	1.81	0.982 (0.65, 1.48)	0.99

Abbreviations: CI—Confidence Interval, MRSA–Methicillin-resistant *Staphyloccocus aureus*, VRE—Vancomycin-resistant *Enteroccoccus*

^a^ Calculated by poisson distribution method

^*b*^ Calculated by chi-square test

**Table 4:** Mortality in the 2 years under study.

Mortality	Year 2012 N = 1183^a^ n (%)	Year 2014 N = 1112^a^ n (%)	p-value^d^
All-cause deaths	131 (11)^b^	98 (9) ^b^	0.12
Bacteraemia-related deaths	9 (8.7) ^c^	6 (6.8) ^c^	0.65

^a^Total number of unique patients admitted in respective year.

^b^Expressed as percentage of total number of unqiue patients admitted

^c^Expressed as a percentage of total all-cause deaths

^*d*^Calculated by chi-square test

**Table 5:** Bacteraemia & antibiotic resistance in Gram-negative strains of blood isolates.

Bacteraemia	Year 2012 N = 2012 n	Year 2014 N = 1843 n	Incidence Rate Ratio (95% CI) ^a^	p-value ^a^
Total, n	104	88	-	-
Total/1000 pt days	3.99	3.62	0.97 (0.68, 1.21)	0.55
Polymicrobial, n	10	4	-	-
Polymicrobial/1000 pt days	0.38	0.16	0.43 (0.14, 1.37)	0.23
Monomicrobial, n	94	84	-	-
Monomicrobial/1000 pt days	3.60	3.45	0.96 (0.71, 1.29)	0.78
Gram-negative, n	55	57	-	-
Gram-negative/1000 pt days	2.11	2.34	1.11 (0.77, 1.61)	0.58
**Gram-negative** **blood isolates**	**n**	**n**	**p-value** ^**a**^	
*E*. *coli*	25	32	0.08	
*Klebisella spp*.^c^	14	13	0.83	
*Enterobacter spp*^*d*^	5	2	0.45	
*P*. *aeruginosa*	11	10	1.00	
Total	55	57	0.58	
**Antibiotic resistance in Gram-negative blood isolates**	**n (%)**	**n (%)**	**p-value** ^**b**^	
**Cef Resistance**				
*E*. *coli*—Cef R	12 (48.0) ^e^	18 (56.3) ^e^	0.60	
*Klebsiella spp*.—Cef R	11 (78.6) ^f^	9 (69.2) ^f^	0.68	
*Enterobacter spp*.—Cef R	0 (0) ^g^	2 (100) ^g^	**0.048**	
*P*. *aeruginosa*—Cef R	6 (54.5) ^h^	3 (30.0) ^h^	0.39	
Total—Cef R	29 (52.7%) ^i^	32 (56.1%) ^i^	0.85	
** ****PTZ Resistance**	**n (%)**	**n (%)**	**p-value** ^**b**^	
*E*. *coli*—PTZ R	12 (48.0)^e^	21 (65.6) ^e^	0.82	
*Klebsiella spp*.—PTZ R	11 (78.6)^f^	10 (76.9) ^f^	1.00	
*Enterobacter spp*.—PTZ R	5 (100)^g^	2 (100) ^g^	1.00	
*P*. *aeruginosa*—PTZ R	4 (36.4) ^h^	2 (20) ^h^	0.64	
Total–PTZ R	32 (58.2%) ^i^	35 (61.4%) ^i^	0.85	
** ****Carbapenem Resistance**	**n (%)**	**n (%)**	**p-value** ^**b**^	
*E*. *coli*—carbapenem R	0 (0) ^e^	0 (0) ^e^	NA	
*Klebsiella spp*. -carbapenem R	0 (0) ^f^	1 (7.7) ^f^	0.48	
*Enterobacter spp*.*—*carbapenem R	0 (0) ^g^	0 (0) ^g^	NA	
*P*. *aeruginosa—*carbapenem R	7 (63.6) ^h^	9 (90.0) ^h^	0.31	
Total—carbapenem R	7 (12.7%) ^i^	10 (17.5%) ^i^	0.60	

Abbreviations: Pt–patient, Cef–cefepime, R–resistant, PTZ–piperacillin-tazobactam, CI–confidence interval

^a^ Calculated by poisson distribution method

^b^ Calculated by chi-square test

^c^ Refers to all bacteria identified under the genus Klebsiella

^d^ Refers to all bacteria identified under the genus Enterobacter

^e^ Expressed as a percentage of all E. coli blood isolates for the corresponding year

^f^ Expressed as a percentage of all Klebsiella blood isolates for the corresponding year

^g^ Expressed as a percentage of all Enterobacter blood isolates for the corresponding year

^h^ Expressed as a percentage of all P. aeruginosa blood isolates for the corresponding year

^i^ Expressed as a percentage of all gram-negative blood isolates for the corresponding year

## Discussion

In this report, we demonstrate the clinical effects of subjecting a population of patients to a strategy of “mixing”. This is the term used by Bergstrom *et al* when one half of treated patients are assigned to one drug, and the other half to another. (11) Bergstrom *et al* show that the proportion of patients colonized with resistant organisms is greater for any cycling interval compared with 50–50 mixing. (11) Our day-by-day alternation of empirical first-line antibiotics is the closest strategy to the 50–50 mixing envisaged by Bergstrom *et al*.

Most important, the strategy was safe. The primary outcome, all-cause mortality, was unchanged in the two observation periods. (Table 4)

We could not run this as a randomized controlled trial, which is the major limitation. Non-consenting patients would mean that the strategy could not be assessed, as the strategy of alternating antibiotics targeted the ecology of the ward. We were unsure if the strategy would be followed in the wards. Mixed results from other studies were attributed, in part, to poor compliance. [19] Hence, though the decision to alternate was reached in mid-2013, we collected data in this “before-after” study for the “after’ period beginning in January 2014 in order to allow sufficient time for habits to change. Intermediate outcomes (e.g. use of antibiotics other than the first-line ones) could not be controlled. The data show that staff were compliant with the guidelines. CEF use almost halved, and PTZ use almost doubled in the “after” period, indicating the shift towards the new guideline. (Table 2) We also could not control the patient mix (e.g. through block randomization). Indeed, there were fewer patients with a new diagnosis of AML in 2014. But, the parameter that mattered–the number of new AML patients who started on chemotherapy–was not significantly different between the two years being studied. (Table 1)

At least two other groups have attempted to introduce antibiotic heterogeneity with encouraging outcomes. The studies of Sandiumenge *et al* were restricted to an ICU setting. [14, 15] They compared resistance rates between periods during which antibiotics were “scheduled” (certain antibiotics were “prioritized” and others “restricted”), “patient-specific” (i.e. given according to patient characteristics), or “mixed” (given to consecutive patients in pre-determined fashion). In both studies, they were able to achieve a high AHI. In the first study, the rate of carbapenem-resistant *Acinetobacter baumannii* fell during the mixing period. [14] In the second study, the authors concentrated on the rates of the ESKAPE organisms in patients with ventilator-associated pneumonia (VAP). [15] The main finding was that the incidence of resistant-ESKAPE organisms causing VAP fell during the mixing period.

Takesue *et al* adopted a different approach to try to achieve heterogeneity. [16] Their study was not restricted to the ICU. They classified antibiotics into “restricted” or “recommended” or “non-supervised” (free-to-use) categories according to resistance rates to that class of antibiotic in the previous 3 months. They also achieved an increase in AHI, and the rate of resistant *P*. *aeruginosa* fell during the intervention period.

To our knowledge, our study represents the first attempt to bring about antibiotic heterogeneity in a Haematology setting. The AHI did rise after the alternate-day strategy was introduced, but not to levels achieved by Takesue *et al* or Sandiumenge *et al*. It is likely that the range of antibiotics used in the Haematology ward is not as broad as in other wards. Many patients are on a quinolone as it is our policy to use quinolones for febrile neutropenia prophylaxis. Amoxicillin-clavulanate and ceftriaxone are hardly used since, with febrile neutropenia in inpatients, only anti-pseudomonal antibiotics are recommended. [1]

The lack of a statistically significant difference in mortality and bacteraemia rates in the two years under study suggests that the mixing strategy was safe. (Tables 4 & 5) Strikingly, the combined use of gram-positive agents fell in the mixing period. (Table 2) The rates of newly-acquired MRSA and VRE were unchanged in the two periods. (Table 3) There were 2 cases of VRE bacteraemia in 2012, and 3 in 2014. We therefore surmise that there were fewer fevers that persisted beyond the 48-hour mark. This squares with our anecdotal observations, as vancomycin tends to be an add-on drug when fever persists. This might indirectly indicate that the infections that triggered the fevers responded better to the antibiotics started in the mixing period. The lower overall antibiotic use, represented by a lower DDD after the alternate-day strategy was implemented, provides support for this theory. (Table 2)

We were not able to show a difference in the rates of new cases of VRE or MRSA. Of the preventive measures against MRSA, the effective ones include improved hand hygiene, active surveillance followed by decolonization, and single-room isolation.[20] As single rooms are scarce in our hospital, we only isolate certain categories of MRSA- and VRE-positive patients. Antibiotic manipulation does not appear to be as crucial in the control of these pathogens.

However, the CRE rate appeared not to have increased in our unit, despite a big increase hospital-wide. Papers describing attempts at CRE control have reported bundled interventions, making it difficult to tease out the component that worked.[21] However there is a general consensus that lowering the overall volume of antibiotics might help with CRE containment. [21] The statistically significant overall DDD after implementation of the alternate-day strategy might hence have played a role. The unchanged rate of new cases of MRSA and VRE suggests that infection control practices were consistent across the time periods.

## Conclusion

In summary, alternating first-line antibiotics in febrile neutropenia in patients with hematologic malignancies resulted in an increase in antibiotic heterogeneity, without increasing mortality. There was also no significant increase in bacteraemia rates and compliance with the use of the strategy was excellent.

## Supporting information

S1 Appendix Laboratory methods. (DOCX)
