# Supplementary material for: Impact of an alternating first-line antibiotics strategy in febrile neutropenia
Source: PLoS One. 2018 Nov 28;13(11):e0208039. doi: 10.1371/journal.pone.0208039 (PMC6261621; doi:10.1371/journal.pone.0208039)
Supplement: S1 Appendix — (DOCX) [file pone.0208039.s001.docx]

**S1 Appendix**

## Laboratory Methods

### Blood Cultures

Blood culture bottles are incubated in the BACTEC FX machines. The aerobic and anaerobic bottles are incubated at 35^O^C for 5 days, while the fungal bottles are incubated at 35^O^C for 14 days. Aerobic bottles that are flagged positive are sub-cultured onto Trypticase soya agar with 5% sheep blood agar and MacConkey agar and incubated at 35^O^C in 5% carbon dioxide. Anaerobic bottles that are flagged positive are sub-cultured onto Trypticase soya agar with 5% sheep blood agar, and MacConkey agar which are incubated at 35^0^C in 5% carbon dioxide, and CDC anaerobic blood agar incubated at 35^O^C anaerobically. Fungal bottles that are flagged positive are sub-cultured onto Trypticase soya agar with 5% sheep blood and incubated at 35^0^C in 5% carbon dioxide, and Brain Heart Infusion agar, Sabouraud Dextrose agar, Sabouraud Dextrose agar with chloramphenicol and Brain Heart Infusion agar with chloramphenicol and gentamicin which are incubated at 35^0^C in ambient air. Bottles that are not flagged are reported as negative upon the end of the incubation cycle.

Positive isolates were identified using biochemical tests until March 2013, following which the Matrix-assisted laser desorption/ionization (MALDI) was introduced for use for bacterial and yeast identification. Susceptibility testing is performed using disk diffusion and interpreted using Clinical and Laboratory Standards Institute (CLSI) interpretive guidelines.

### MRSA Screening

Swabs received for MRSA culture are inoculated into Brain Heart Infusion broth with 6mg/L oxacillin and incubated at 35^0^C for 18 to 28 hours. The broth is then sub-cultured onto MRSA *Select* II agar (Bio-Rad) and incubated at 35^0^C for 18 to 28 hours. Pink-coloured colonies are picked and latex agglutination using Pastorex Staph-Plus kit (Bio-Rad) is performed. Pink colonies that are latex agglutination positive are reported as positive for MRSA.

### VRE Screening

Swabs received for VRE screening are inoculated onto Enterococcosel Agar with 6µg/ml of Vancomycin (Thermo Fisher Scientific). The plates are incubated at 35^0^C for up to 72 hours. Suspicious colonies are identified using biochemical tests from 2012 until March 2013, and replaced with the MALDI from April 2013. The susceptibility is performed using disk diffusion and Vitek 2 cards.

### CRE Screening

Swabs received for CRE screening are inoculated onto chromID CARBA Agar (bioMérieux SA, France) from April 2013. The plates are incubated at 35^o^C for up to 48 hours. Suspicious colonies are identified using MALDI. Susceptibility testing is performed using disk diffusion as well as the Etest method. Modified Hodge Test is used to detect carbapenemase production. Phenotypic confirmation of carbapenemase-producing (CP)-CRE is performed using the ROSCO-KPC, Metallo-beta-lactamase and OXA-48 Confirmation Kit (Rosco Diagnostica). From August 2013, CP-CRE genotyping was introduced and used on top of the phenotypic tests.
